# The Role of TG2 in Regulating S100A4-Mediated Mammary Tumour Cell Migration

**DOI:** 10.1371/journal.pone.0057017

**Published:** 2013-03-01

**Authors:** Zhuo Wang, Martin Griffin

**Affiliations:** School of Life and Health Sciences, Aston University, Aston Triangle, Birmingham, United Kingdom; King's College London, United Kingdom

## Abstract

The importance of S100A4, a Ca^2+^-binding protein, in mediating tumour cell migration, both intracellularly and extracellularly, is well documented. Tissue transglutaminase (TG2) a Ca^2+^-dependent protein crosslinking enzyme, has also been shown to enhance cell migration. Here by using the well characterised non-metastatic rat mammary R37 cells (transfected with empty vector) and highly metastatic KP1 cells (R37 cells transfected with S100A4), we demonstrate that inhibition of TG2 either by TG2 inhibitors or transfection of cells with TG2 shRNA block S100A4-accelerated cell migration in the KP1cells and in R37 cells treated with exogenous S100A4. Cell migration was also blocked by the treatment with the non-cell permeabilizing TG2 inhibitor R294, in the human breast cancer cell line MDA-MB-231 (Clone 16, which has a high level of TG2 expression). Inhibition was paralleled by a decrease in S100A4 polymer formation. *In vitro* co-immunoprecipitation and Far Western blotting assays and cross-linking assays showed not only the direct interaction between TG2 and S100A4, but also confirmed S100A4 as a substrate for TG2. Using specific functional blocking antibodies, a targeting peptide and a recombinant protein as a competitive treatment, we revealed the involvement of syndecan-4 and α5β1 integrin co-signalling pathways linked by activation of PKCα in this TG2 and S100A4-mediated cell migration. We propose a mechanism for TG2-regulated S100A4-related mediated cell migration, which is dependent on TG2 crosslinking.

## Introduction

The onset of tumour metastasis is a complicated process involving complex intracellular cell signalling network(s) elicited via cell contact with the extracellular matrix (ECM), and also by crosstalk between tumour cells, stromal cells and immune cells. One important protein involved in this crosstalk is S100A4. S100A4 is a member of the Ca^2+^-binding protein S100 family, which has been widely found to be over-expressed in highly metastastic cancers and characterized as a marker of tumour progression [1], [2]. S100A4 is reputed to act both in the intracellular and extracellular environment. Intracellular S100A4 can bind directly to the myosin light chain to mediate cytoskeletal organization and in turn promote cell migration [3]. Via its direct interaction with NF-κB, S100A4 is also reputed to be involved in cancer cell proliferation and differentiation [4]. However, S100A4 is also found in the extracellular environment, where it can be externalised from cancer cells and surrounding stromal and immune cells via an unknown non-coventional secretion pathway. Extracellular S100A4, like the intracellular protein, can also promote cell migration, but its mode of action is still not fully undertsood. It has been suggested that RAGE [5] or α6β4 integrin [6] could be the cell surface receptors involved in transducing the S100A4-mediated signalling, while other research suggests the involvement of cell surface heparan sulphates in the signal transduction process [7].

Another important protein, which functions both in the intra- and extracellular environment and which is linked to cancer progression both in breast and other cancers, is the multifunctional enzyme tissue transglutaminase (TG2) [8]. Like S100A4, TG2 is a Ca^2+^-binding protein, which mediates a transamidating reaction leading to protein crosslinking in a Ca^2+^-dependent manner [9]. In the intracellular environment, its transamidation activity is tightly regulated by the binding of GTP/GDP, but its activity is easily detectable at the cell surface or in the extracellular matrix, where activating levels of Ca^2+^ are available [9]. In adition, cell surface TG2 may act extracellularly as a novel adhesion protein via it its binding to fibronectin (FN) and association with β1 and β3 integrins [10] and with cell surface heparan sulphates [11]–[13]. It has also been shown that, in breast cancer cells, TG2 may function as a scaffold protein via its potential association with the actin cytoskeleton [14]. Importantly in many cancer cells increased TG2 activity is associated with an increased malignant phenotype including increased drug resistance, which can be reversed by TG2 siRNA silencing [15]. Through an unknown secretion pathway, TG2, like S100A4, is externalized onto the cell surface and into the ECM, where like S100A4 it has been shown to bind to cell surface heparan sulphates for which it has a high affinity and which are thought necessary for translocation of the enzyme into the ECM [12]. Cell surface heparan sulphates are also required for maintaining its transamidation activity and the function of TG2 as a cell adhesion protein [11], [13]. We recently reported that syndecan-4, a member of the heparan sulphate proteoglycan family, can via its binding to TG2 mediate a novel RGD-independent cell adhesion mechanism involving activation of PKCα and activation of α5β1 integrin. The inside-out signalling mechanism which is elicited is also able to enhance the formation and deposition of FN fibrils [16].

Even though there is no direct link between TG2 and S100A4-mediated cell migration, it has been shown that TGs, including TG2, can crosslink members of the S100 family, such as S100A7, S100A10 and S100A11 [17]. Interestingly, the mutagenesis of the C-terminus of S100A4, which is the target for TG2 crosslinking prevents the enhanced migratory phenotype. Given this close link between the S100A4 protein and TG2 and their ability to affect cell cancer cell migration our objective in this paper was to explore the potential involvement of TG2 in regulating S100A4-related cell migration and to elucidate the mechanisms involved.

## Materials and Methods

### Reagents and Antibodies

Human plasma FN and recombinant human S100A4 were from Sigma-Aldrich (UK) and R&D system (UK), respectively. Purified guinea pig liver TG2 (gplTG) was purified according to Leblanc et al [18]. Unit of enzyme activity is defined as the formation of 1.0 μmol of hydroxamate per min from Nα-Z-Gln-Gly and hydroxylamine at pH 6.0 at 37°C. Vectashield mounting medium was purchased from Vector Laboratories. Rat TG2 shRNAs and the control shRNA were purchased from Qiagen (UK). The cell culture medium solutions and antibiotics were from PAA (UK). Lipofectamine 2000 reagent and the OptiMEM medium were from Invitrogen (UK). The site-directed irreversible transglutaminase inhibitors 1,3-dimethyl-2 imidazolium derivative R283 and the peptidic inhibitors R294 and R281 were synthesized at Aston University, as previously described [19]. The TG2 specific inhibitor Z-DON (Z-DON-Val-Pro-Leu-OMe) was purchased from Zedira (Germany). XTT reagents were from Roche (UK).

The commercial antibodies used in this study are listed in **Table 1**. The HRP-conjugated anti-mouse or rabbit secondary antibodies were purchased from Sigma-Aldrich (UK) and Dako (Denmark), respectively. Mouse monoclonal antibody 1D10 raised against gplTG was manufactured in house.

**Table 1:** List of antibodies used for IHC, IF and Western blotting.

Antigen	Species source	Company
TG2 (Cub7402)	Mouse, monoclonal	Neomarkers (Thermo, UK)
TG2 (TG100)	Mouse, monoclonal	Neomarkers (Thermo, UK)
TG2	Rabbit, polyclonal	Cell Signalling (NEB, UK)
S100A4	Mouse, monoclonal	AbD Serotec (UK)
S100A4	Rabbit, polyclonal	Dako (Denmark)
TG1	Rabbit, polyclonal	Abcam (UK)
Syndecan-4	Rabbit, polyclonal	Invitrogen (UK)
β1 Integrin	Rabbit, polyclonal	Santa Cruz (UK)
β4 Integrin	Rabbit, polyclonal	BD biosciences (UK)
α5 Integrin	Rabbit, polyclonal	Santa Cruz (Germany)
α6 Integrin	Rabbit, polyclonal	Cell Signalling (NEB, UK)
RAGE	Rabbit, polyclonal	Cell Signalling (NEB, UK)
PKCα	Mouse, monoconal	Santa Cruz (UK)
Caspase-3	Rabbit, polyclonal	BD Biosciences (UK)
6×His tag	Mouse, monoconal	Invitrogen (UK)
α-Tubulin	Mouse, monoconal	Sigma-Aldrich (UK)

### Cell lines

The well characterised rat mammary cell lines [20] used in this study were a king gift from Dr. Roger Barraclough (University of Liverpool, UK) and consisted of a non-metastatic rat mammary cells Rama37 stably transfected with either a Neo-Vector (R37) or plasmid containing human S100A4 (KP1). Cells were selected by using G418 to establish the stable cell lines. The cells were maintained in complete DMEM medium containing G418 as described previously [20]. The MDA-MB-231 (Clone 16), which has high TG2 expression and shows increased migration and increased resistance to doxorubicin, was a kind gift of Dr. Kapil Mehta (University of Texas, USA). The MDA-MB-231 wild type cell line was a kind gift from Dr. Stephane Gross (Aston University, UK).

To knock down TG2 expression, R37 and KP1 cells were transfected with the rat TG2 shRNA (#1–4) or the control scrambled shRNA using Lipofectamine 2000 reagent following the manufacture's instruction and the transfected cells were selected using puromycin (5 μg/ml) after 48 h transfection. The stable cell lines were maintained in cell culture medium containing 2.5 μg/ml puromycin.

### Cleavage of the 6× his-tag from the recombinant proteins

The cleavage of the 6× his-tag from the recombinant S100A4 protein was performed using the Enterokinase cleavage capture kit (Novagen, UK) following the manufacture's instruction. After optimization, the rEK: target protein ratio at 1∶50 was chosen to remove the his-tags from the recombinant S100A4 at room temperature for 16 h. The recombinant Enterokinase was removed using the EKapture Agarose columns and the target protein was recovered for future experiments.

### *In vitro* crosslinking

*In vitro* crosslinking assays were performed as documented previously [17]. Briefly, 1 μg of recombinant human S100A4 protein was incubated with gplTG (0.05-1U) in the presence of 10 mM CaCl_2_ for 1 h at 37°C in reaction buffer containing 50 mM Tris-HCl, pH 7.4, 1 mM EDTA, 3.4 mM DTT, 30 mM NaCl, 0.1% Triton X-100. The reaction was terminated by adding 20 mM EDTA and by incubating the samples on ice. SDS-PAGE and Western blotting were used to detect the presence of S100A4 polymers by using anti-S100A4 antibody.

### Co-immunoprecipitation

Co-immunoprecipitation (co-IP) assays were performed following incubation of 1 μg of recombinant human S100A4 and 1 μg of gplTG at 37°C for 1 h. For the interaction between syndecan-4 and crosslinked S100A4, the *in vitro* crosslinking reaction was performed as introduced above in the presence or absence of TG inhibitor R283, followed by incubating the products with 1 µg of recombinant human syndecan-4 at 37°C for 1 h. The co-IP assay was performed by using the co-IP kit (Pierce, UK) according to the manufacture's instruction. Briefly resin beads were coated with the relevant antibodies (i.e. anti-S100A4, anti-TG2 or anti-syndecan-4 antibody). The immunocomplexes were incubated with the beads, then collected into Laemmli sample buffer and then boiled at 100°C for 5 min. Western blotting was carried out by using specific antibodies to detect the targetting antigen in the immunocomplexes as introduced previously [16].

### Far Western blotting

This assay was performed as documented previously [21]. S100A4 or TG2 was blotted onto Immobilon-P membranes. Membranes were then blocked using 50 mM Tris-HCl, pH 7.5, containing 0.2 M NaCl, 3% bovine serum albumin (BSA) and 0.1% polyethylene glycol 8,000 for 2 h at room temperature (blocking buffer). 1 μg of the bait proteins (TG2 for S100A4 membranes and S100A4 for TG2 membranes) were then incubated with the membranes for 20 min at room temperature in the reaction buffer (blocking buffer plus 1 mM CaCl_2_). After washing three times, Western blotting was performed to detect the bait protein on the membrane.

### Dot blotting

R37 and KP1 cell culture medium was collected and the dot blotting and Western blotting for the presence of S100A4 was performed as described previously [12].

### Solid phase binding assay to study the interaction of S100A4 with TG2

Recombinant human S100A4 (after removal of the his-tags, at 1 μg/ml in Na_2_CO_3_ solution, pH 9.6) was coated onto 96- well plates at 4°C overnight. After washing three time with 50 mM Tris-HCl, pH 7.4, the wells were blocked with 3% BSA in PBS, pH 7.4, 2 μg/ml recombinant human syndecan-4 (with his-tag) in 50 mM Tris-HCl, pH 7.4, was incubated in the wells at 37°C for 1 h. The presence of syndecan-4 bound to the S100A4-coated wells was detected by using anti-his-tag antibody and anti-mouse secondary antibody. The signals were detected using OPD substrate and the absorbance measured at 490 nm.

### TG activity assay

The commercial TG2-CovTest (Covalab) specific for TG2 activity in cell lysates was performed according to the manufacture's instruction [22]. Briefly, the cell lysate containing 50 µg of total protein was incubated with spermine pre-coated wells in the presence of biotin-labelled TG2 specific T26 peptide, DTT and Ca^2+^. The incorporated T26 peptide was detected by incubating with HRP-conjugated Extr-avidin. The reaction was developed using OPD substrates and measured at 450 nm by using a plate reader.

Cell surface TG activity was measured by incubating live cells (2×10^4^/well) with 0.1 mM biotin-cadaverine in serum free medium on FN-coated wells at 37°C for 2 h. The reaction was stopped with 2 mM EDTA in PBS, pH 7.4, and the cells were removed by 0.1% (w/v) deoxycholate in 2 mM EDTA in PBS, pH 7.4. The rest of the reaction was performed as described above for cell lysates above using HRP-conjugated Extr-avidin.

### Wound healing assay

R37 and KP1 cells were grown in complete medium containing 10% FBS in 12-well plates for 48 h to form a mono-cell layer before the wound was scratched. The cells were allowed to migrate in medium containing 1.5% serum (in order to limit any interference on migration by cell proliferation) at 37°C for 16 h in the presence of the different treatments. The cells were fixed with 3.7% paraformaldehyde in PBS, pH 7.4 for 15 min and permeabilized with 0.1% Triton in PBS, pH 7.4 for 15 min. May-Graunward and Giemsa co-staining was performed as described previously [23]. The images of the wound areas were taken by using Nikon digital camera at ×20. At least 3 images were taken from each wound and the closure of the wound areas was calculated. R37 cells without the migratory ability were used as the control group. The wound areas were measured using the ImageJ software and the percentage closure of the wound areas presented as mean ± S.D. from 3 separate experiments.

### Fluorescence staining

For staining of the extracellular S100A4 and *in situ* active TG2 in KP1 cells, the cells were pre-incubated with 8 μM FITC-cadaverine in the presence or absence of non cell-permeable TG2 inhibitor R294 in complete medium at 37°C for 6 h [24]. To detect the extracellular S100A4, rabbit anti-human S100A4 antibody (1∶100 dilution) was then added into the cells for another 2 h incubation at 37°C. After washing three times carefully with PBS, pH 7.4, the cells were fixed with methanol at −20°C for 20 min. After washing three times with PBS, pH 7.4 and blocking with 3% BSA in PBS, pH 7.4 (blocking buffer) for 30 min at room temperature, the cells were incubated with TRITC-conjugated anti-rabbit secondary antibody in blocking buffer (1∶100 dilution) at 37°C for 2 h.

For vinculin and active form of caspase-3 staining KP1 cells were fixed with 3.7% paraformaldehyde in PBS, pH 7.4 and permeabilized with 0.1% Triton in PBS, pH 7.4. Cells were then incubated for 4 h with specific primary antibodies and FITC or TRITC-conjugated secondary antibody (1∶100 dilution in blocking buffer) for 2 h/incubation. For the actin cytoskeleton staining, 15 μg/ml FITC-phalloidin was used in blocking buffer for 2 h at 37°C.

Following staining, cells were mounted in Vectorshield mountant medium and the fluorescence signal detected by fluorescence microscopy.

### Cell migration assay

Migration assays were undertaken as previously described [24]. R37 and KP1 cells were seeded onto 8 mm cover slips pre-coated with 5 μg/ml FN in 50 mM Tris-HCl, pH 7.4 in 24 well plates and incubated for 1 h in serum-free medium to obtain cell monolayers. The cover slips were then removed and transferred into new 24-well plates coated with 5 μg/ml FN and the migration of cells from the cover slip monitored for 4 h in the presence or absence of the TG inhibitors. The total cell number which migrated from the cover slips into the wells was then counted. Three experiments were performed for each assay.

### Cell viability assay

The XTT assay was used to measure viable cell numbers as described previously [25].

### Cytosol and membrane fractionation

The cytosol and membrane fractions of R37 cells, stably transfected with control scrambled or #3 TG2 shRNA, were isolated as described previously [23]. Briefly cells were treated with exogenous S100A4 in the presence or absence of TG2 inhibitor R294 for 16 h in cell culture medium containing 1.5% serum. Following incubation, the cells were washed once with ice-cold PBS, pH 7.4 and collected into pre-chilled homogenization buffer containing 20 mM Tris–HCl, pH 7.4, 10 mM EGTA, 2 mM EDTA, 1 mM NaF, 1 mM Na_3_VO_4_, 50 nM okadaic acid, 0.1 mM PMSF and 1% (v/v) protein inhibitor cocktail. The cells were lysed by 4 cycles of freezing and thawing (one cycle includes incubating in liquid nitrogen for 2 min and incubating in water bath at 37°C for 7 min). The cell lysates were then pre-cleared at 1,300×g for 10 min. The supernatants were collected and centrifuged at 100,000×g for 60 min to separate cytosolic (supernatant) and membrane fractions. The membrane fractions were further washed once with ice-cold PBS, pH 7.4. The cytosol and membrane fractions of samples were Western Blotted for PKCα as described above.

### Statistics

Data were expressed as mean ± S.D. In some cases, the data shown is derived from one representative experiment (n>3) undertaken. The comparisons between the data sets were performed using Student's t test (two-tailed distribution with equal variance). Statistical significant difference between data sets is defined in the text by p<0.05 (two-sided).

## Results

### S100A4-transfection enhances cell migration in Rama 37 cells, which can be blocked by TG inhibitors

**Figure 1:**
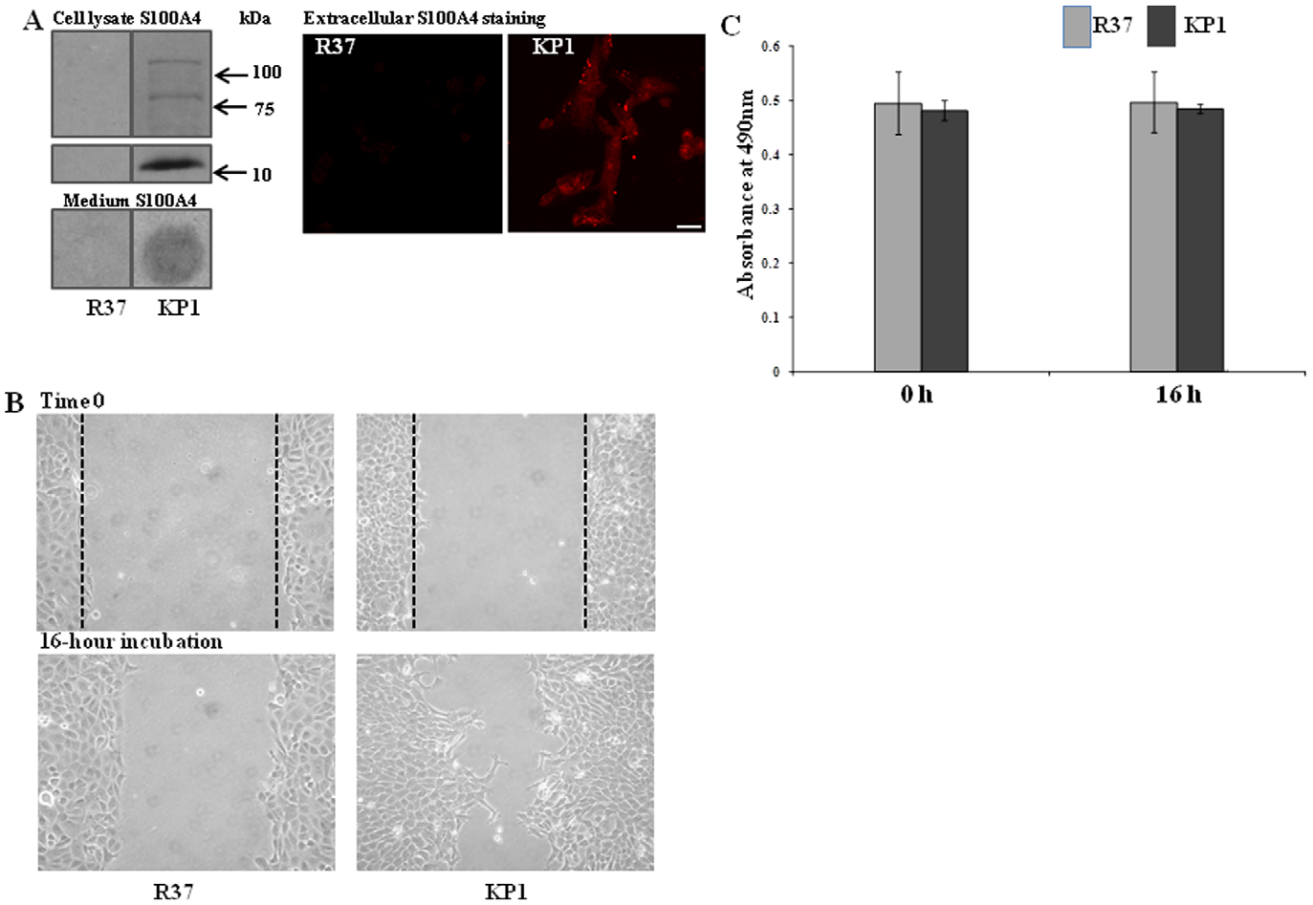
S100A4-transfection enhances cell migration in Rama 37 cells, which can be blocked by TG inhibitors. (**A**) **The presence of S100A4 in R37 and KP1 cells.** The presence of S100A4 in R37 and KP1 cell lysates was detected via Western blotting, while the antigen in the cell culture medium was detected by dot blotting. Extracellular S100A4 was detected by immunofluorescence using anti-human S100A4 antibody using live cells and visualized using confocal microscope as described in the Materials and Methods. Bar, 25 µm. (**B**) **Effect of S100A4 on cell migration**. 1.25×10^5^ cells/well of R37 or KP1 cells cultured in complete medium containing 10% serum were seeded into 12-well plates and incubated for 48 h to reach confluency. Wound areas were scratched by using 200 μl microtips and the cells then allowed to migrate for 16 h in 1.5% serum, thus limiting cell proliferation. At least 3 images were taken from each wound to verify the closure rate of the wound and three separate experiments were performed. The images represent one typical area of each group. (**C**) **Cell proliferation rates of R37 and KP1 cells in 1.5% serum.** R37 and KP1 cells in DMEM medium containing 1.5% serum were seeded into 96-well plates at the density of 3,000 cells/well. The XTT assay was performed at 0 h and 16 h incubation time points as described in the Materials and Methods. Data represent the mean ± S.D. from 3 separate experiments.

**Figure 2:**
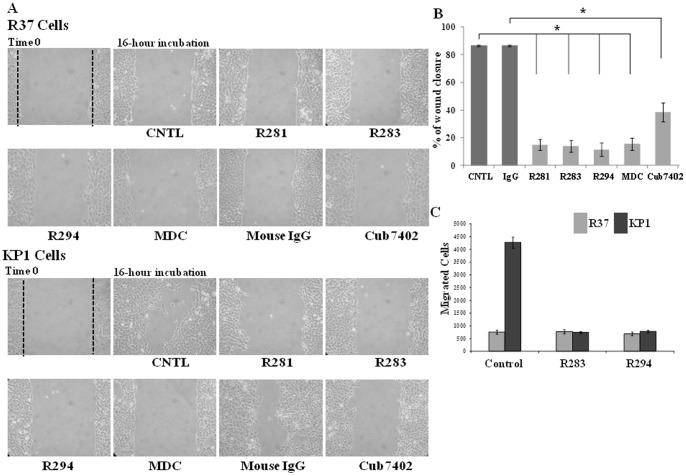
Inhibitory effect of TG2 inhibitors on S100A4-related cell migration. **(A)** Wound healing assays were carried on as introduced in **Fig. 1**. 0.5 mM of TG inhibitors, R281, R283, or 0.25 mM R294, 0.1 mM MDC or TG2-specific antibody Cub7402 (20 μg/ml) was added to the cell culture medium containing 1.5% serum, thus limiting cell proliferation. Wound closure was followed over 16 h. After fixation and staining, the wound areas of the KP1 cells were analysed (**B**) as described in the Materials and Methods. At least 3 separate experiments were performed per treatment. The mean percentage wound closure mean ± S.D. is shown in (**B**). *, p<0.05 between the groups as indicated in the figure. (**C**) **The effect of TG inhibitors on the S100A4-related cell migration in the cover slip migration assay.** R37 and KP1 cells were seeded onto 8 mm FN-coated glass cover slips placed onto FN coated 24-well plates and allowed to migrate for 1 h in serum free medium with or without TG inhibitor R283 or R294. The migrated cells were counted after the staining of the cells as introduced in Materials and Methods. Values represent the Mean values ± S.D. from two separate experiments undertaken in duplicate.

The role of S100A4 in regulating cell migration has been widely reported [1]. A well-reported cell line KP1 (Rama37 cells transfected with S100A4) and its control cell line R37 (Rama37 cell transfected with the empty vector) were used in this study. The cell lines were first characterized via detecting the presence of S100A4 protein in whole cell lysates and cell culture medium via Western blotting and dot blotting, respectively. Immunofluorescent staining was used to detect extracellular S100A4. This confirmed that there was negligible S100A4 in the R37 cells, while S100A4 protein was over-expressed in KP1 cells and was also found to be present in the cell culture medium (**Fig. 1A**). In the cell lysates of KP1 cells large non-reducible Mr polymers could be detected. Using the wound healing assay, enhanced migratory ability was shown in the S100A4 stably-transfected KP1 cells, when compared to their vector transfected control R37 cells, confirming the role of S100A4 in mediating cell migration (**Fig. 1B**). This difference in migration was not due to differences in the cell proliferation rates between these two cells as measured when cultured in the same medium containing 1.5% serum, in which no cell proliferation was observed (**Fig. 1C**). To ascertain whether TG2 activity might also play a role in the migratory ability of KP1 cells, comparable assays were performed in the presence of different TG2 inhibitors. Inhibitors used were either the site directed irreversible inhibitors R283 (cell-permeable) or cell impermeable R281 [26] and R294 [19], the competitive primary amine substrate monodansyl-cadaverine (MDC) and a TG2-specific antibody Cub7402. None of the inhibitors used showed any significant toxicity when used at comparable concentrations to those selected for the wound healing assay **Fig. 1C**. As shown in **Fig. 2** **A** and **B**, all of the inhibitors including MDC and Cub7402 significantly blocked cell migration in KP1 cells. A similar inhibitory effect of TG inhibitors R283 and R294 was also found in the cover slip migration assay which follows the R37 and KP1 cells migrating from the glass cover slip into the cell culture wells in the presence or absence of the inhibitors R283 and R294 (**Fig. 2C**).

### Non-toxic effect of TG2 inhibitors was found in R37 and KP1 cells

**Figure 3:**
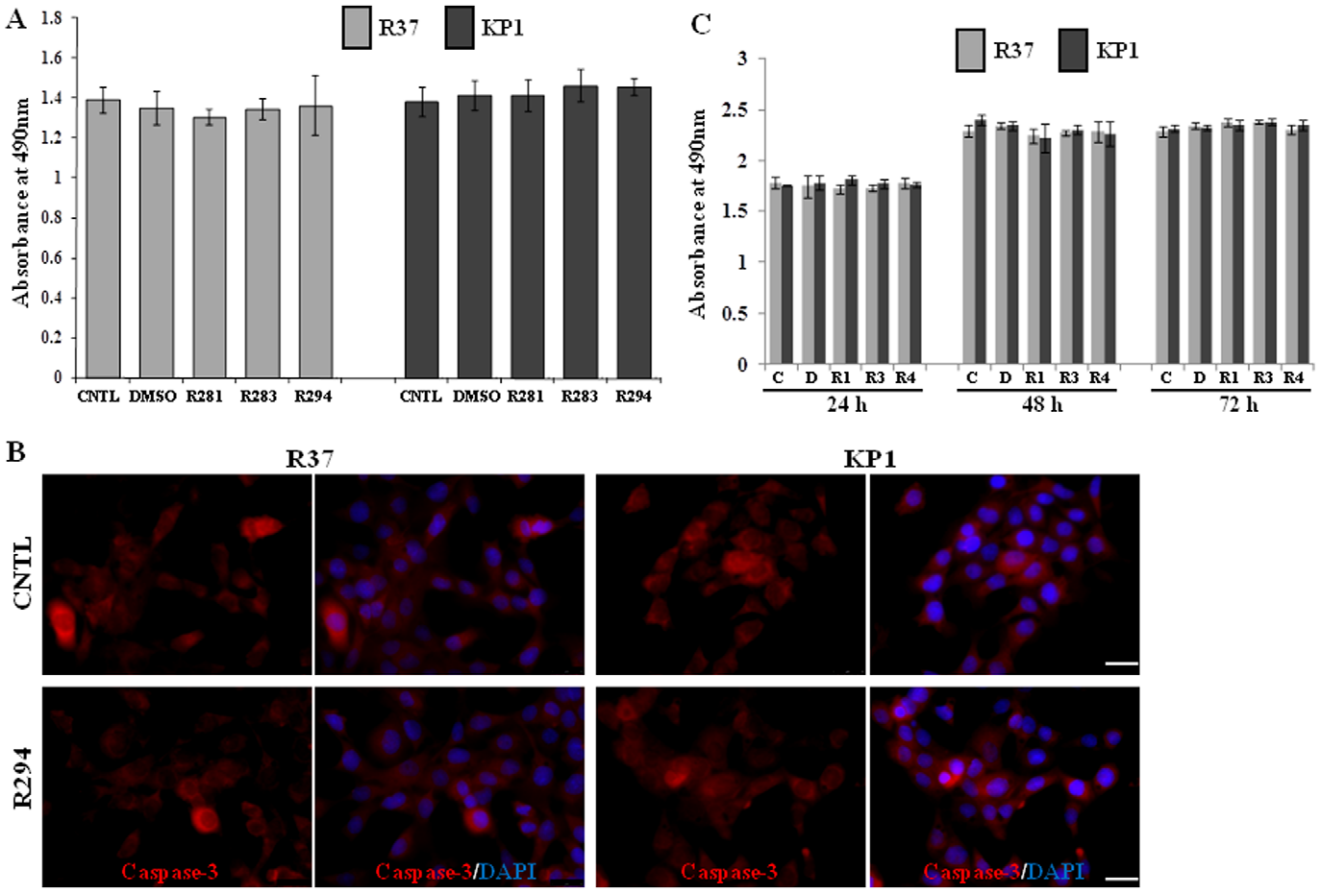
The effect of TG2 inhibitors on R37 and KP1 viability. (**A**) **The effect of TG inhibitors on R37 and KP1 cells using the XTT assay.** R37 and KP1 cells in DMEM medium containing 1.5% serum were seeded into 96-well plates at the density of 3,000 cells/well in the presence or absence of TG2 inhibitors, R281, R283 and R294. The XTT assay was performed after a 16 h incubation time point as described in the Materials and Methods. Data represent the mean ± S.D. from 3 separate experiments. (**B**) **Caspase staining for apoptosis.** The presence of active caspase-3 in the R37 and KP1 cells treated with TG2 inhibitor R294 was detected using fluorescent staining (red) and visualized under a fluorescence microscope as described in the Materials and Methods, while DAPI was used for the nuclei staining (blue). Bar, 25 µm. (**C**) **Effect of TG2 inhibitors on R37 and KP1 cell viability after 72 h.** The TG2 inhibitors were introduced to R37 and KP1 cells at the concentrations described above and incubated upto 72 h. The XTT assays were performed to detect cell viability as introduced in (**A**).

To investigate if there is any toxicity of TG2 inhibitors on R37 and KP1 cells, the XTT assay was undertaken after 16 h incubation with the inhibitors, the time period used for the wound healing assay. As shown in **Fig. 3A**, there was no effect of the TG2 inhibitors on R37 and KP1 cell proliferation. For measurement of apoptosis, the presence of caspase-3 in R37 and KP1 cells was detected via immuno-fluorescence staining. No difference in the fluorescent signal was found between the groups with or without TG2 inhibitor R294 treatment (**Fig. 3B**). When the TG2 inhibitor treatment was extended to 72 h, no effect of the inhibitors on viable cell number was found using the XTT assay (**Fig. 3C**).

### The presence of TG2 and its direct interaction with S100A4 in R37 and KP1 cells

**Figure 4:**
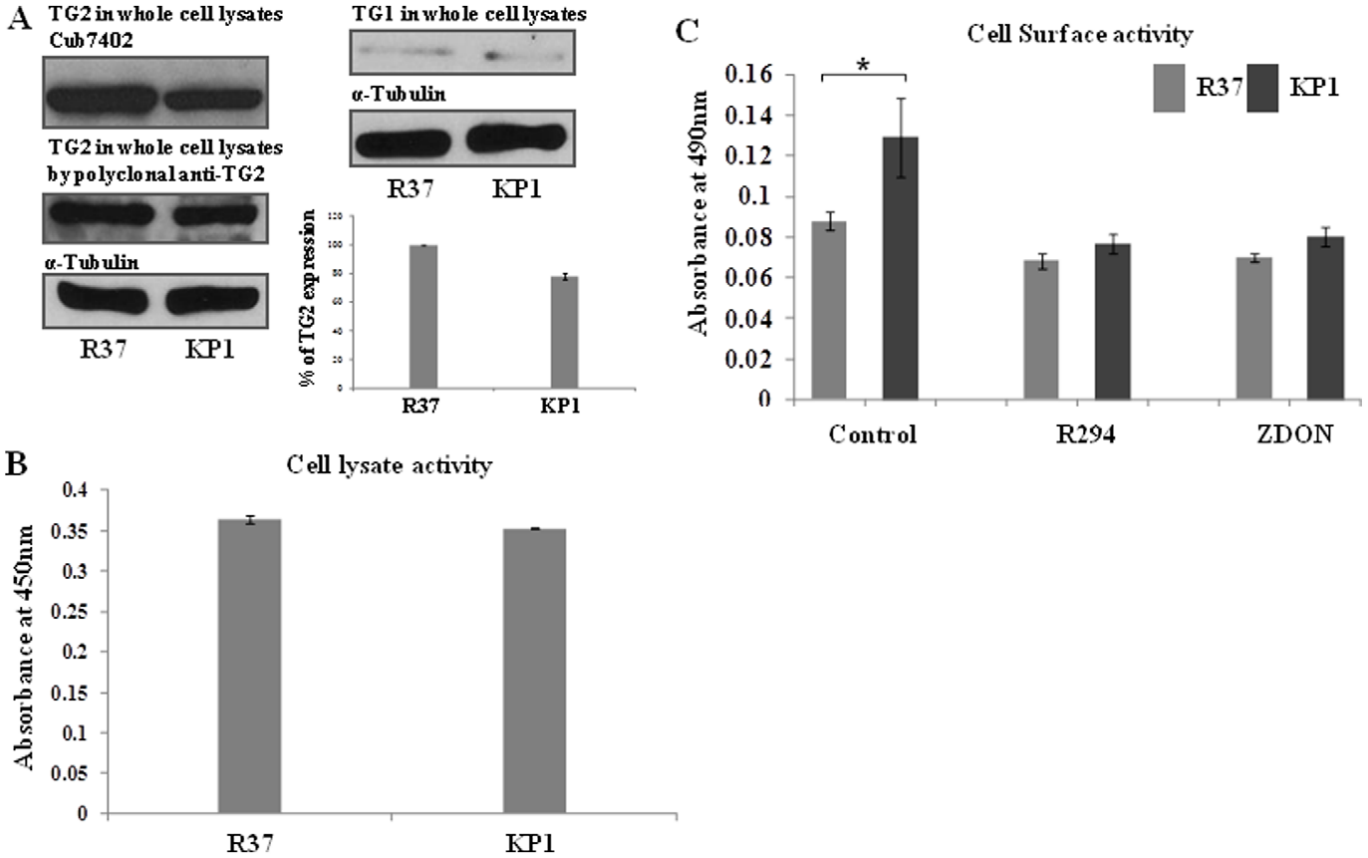
Identification of TG2 in R37 and KP1 cells. (**A**) The presence of TG2 and TG1 in R37 and KP1 cells. Whole cell lysates from R37 and KP1 cells were prepared as described in the Materials and Methods. Western blotting was used to detect the presence of TG2 and TG1 antigen by using the specific monoclonal TG2 antibody Cub7402 and a polyclonal anti-TG2 antibody and a polyclonal antibody against TG1, while α-Tubulin was used as the equal loading standard for densitometric analysis, as shown in the inset histogram (mean ± S.D. from two separate experiments undertaken in duplicate). (**B**) **Detection of TG2 activity in R37 and KP1 cell lysates.** A TG2-specific CovTest assay was performed using R37 and KP1 cell lysates (50 µg protein) as described in the Material and Methods. Data represent mean values ± S.D. from 3 separate experiments. (**C**) Cell surface TG activity was undertaken with live cells (2×10^4^ cells/well). Cells were incubated with biotin-cadaverine in serum free medium for 2 h after plating onto FN coated wells. HRP conjugated Extr-avidin was used to detect the biotin-labelled crosslinking products as described in the Materials and Methods. Data represent mean values ± S.D. from 3 separate experiments. *, p<0.05 compared to the control sample.

To confirm the presence of TG2 in R37 and KP1 cells, Western blotting was performed to detect the antigen in these cells by using both mouse monoclonal anti-TG2 antibody Cub7402 and a rabbit polyclonal anti-TG2 antibody. Easily detectable amounts of TG2 antigen were present in the cell lysates of both R37 and KP1 cells, although higher (approx. 20%) in the R37 cells (Fig. 4A). Also small amounts of the other TG family member, TG1 normally found in cultured epithelial cells could be detected via Western blotting in both cell lines (**Fig. 4A**). The TG2-specific commercial assay for TG2 activity (TG2-CovTest assay) demonstrated activity in the cell lysates of both cells, although as found with Western blots slightly greater in the R37 cells (**Fig. 4B**). Measurement of cell surface activity by biotin-cadaverine incorporation into FN confirmed the presence of cell surface TG2 activity in both cells, but unlike cell lysate activity was found to be significantly greater in the KP1 cells (**Fig. 4C**). Importantly, the TG specific inhibitor R294 (which does not inhibit extracellular Factor XIIIa at the concentration of 0.25 mM) and the TG2 specific inhibitor Z-DON [27]–[29] reduced the cell surface TG activity in both cell lines, suggesting that TG2 is the major cell surface source of the TG activity.

### Knock down of TG2 expression abolishes the migratory capability in KP1 cells

**Figure 5:**
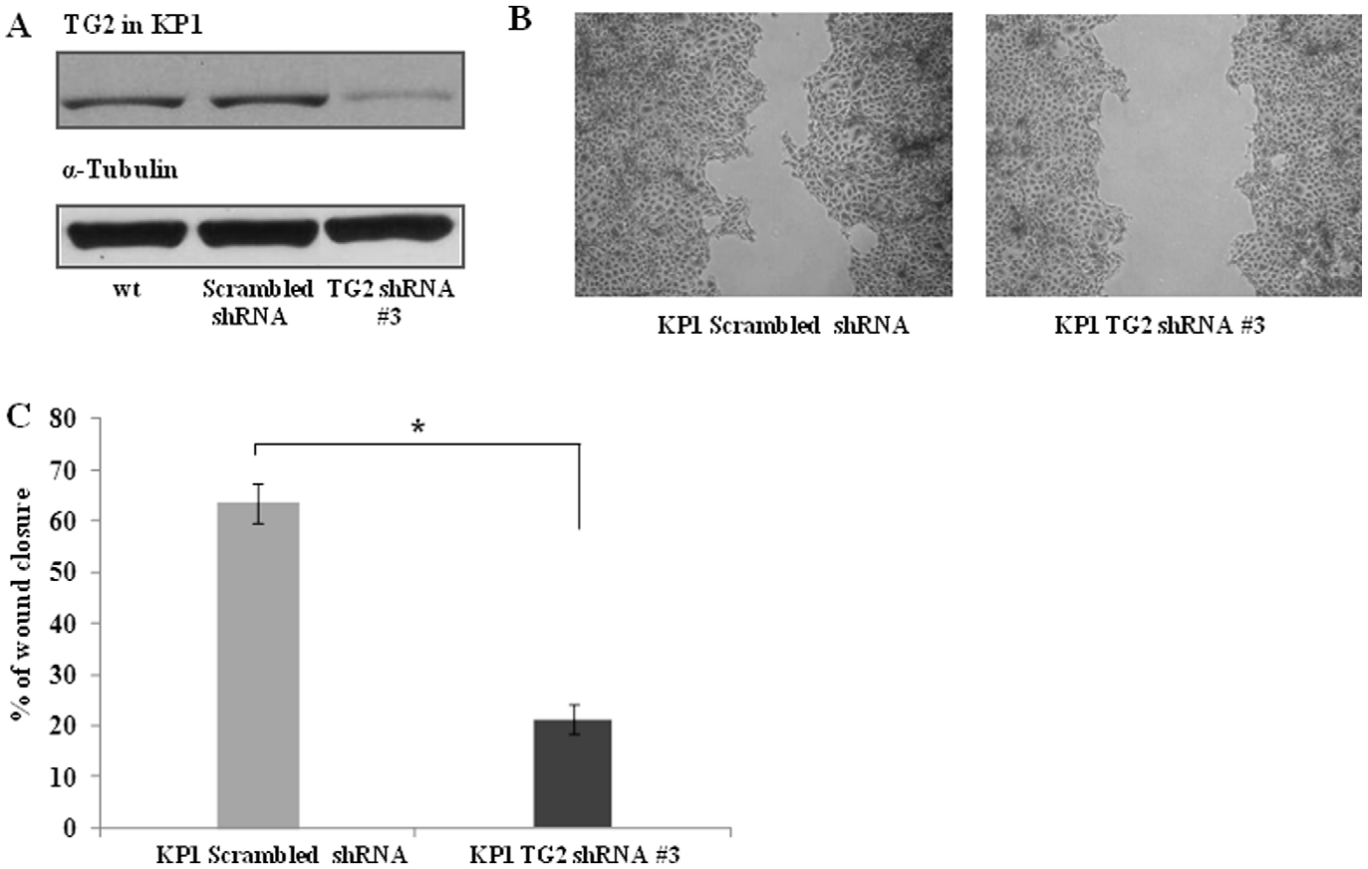
Inhibition of KP1 cell migration by transfection with TG2 shRNA. (**A**) **Knock down of TG2 in KP1 cells.** Western blotting was performed to detect the presence of TG2 in KP1 cells stably transfected with TG2 shRNA (#3), while the scrambled shRNA was used as the control vector. (**B**) **Wound healing assay.** The scratch assay for cell migration was performed as described in Fig. 1B using TG2 shRNA or scrambled shRNA transfected KP1 cells. (**C**) The wound areas of the cells was analysed as described in the Materials and Methods. *, p<0.05 compared to the scrambled shRNA transfected KP1 cells.

TG2 shRNAs were used to knockdown the expression of TG2 in KP1 cells and stably transfected cell lines were generated by puromycin selection. As shown in **Fig. 5A**, shRNA #3 showed the best knockdown in TG2 expression and was used in wound healing assays. No changes in the levels of syndecan-4 or α5 and β1 integrin were present in the shRNA treated cells when compared to their scrambled shRNA controls (**Fig. S1**). Down-regulation of TG2 expression significantly reduced the migratory ability of KP1 cells (**Fig. 5B and C**), confirming the effect of TG2 inhibition on the migratory ability of KP1 cells.

### S100A4, a substrate of TG2, directly interacts with the enzyme

**Figure 6:**
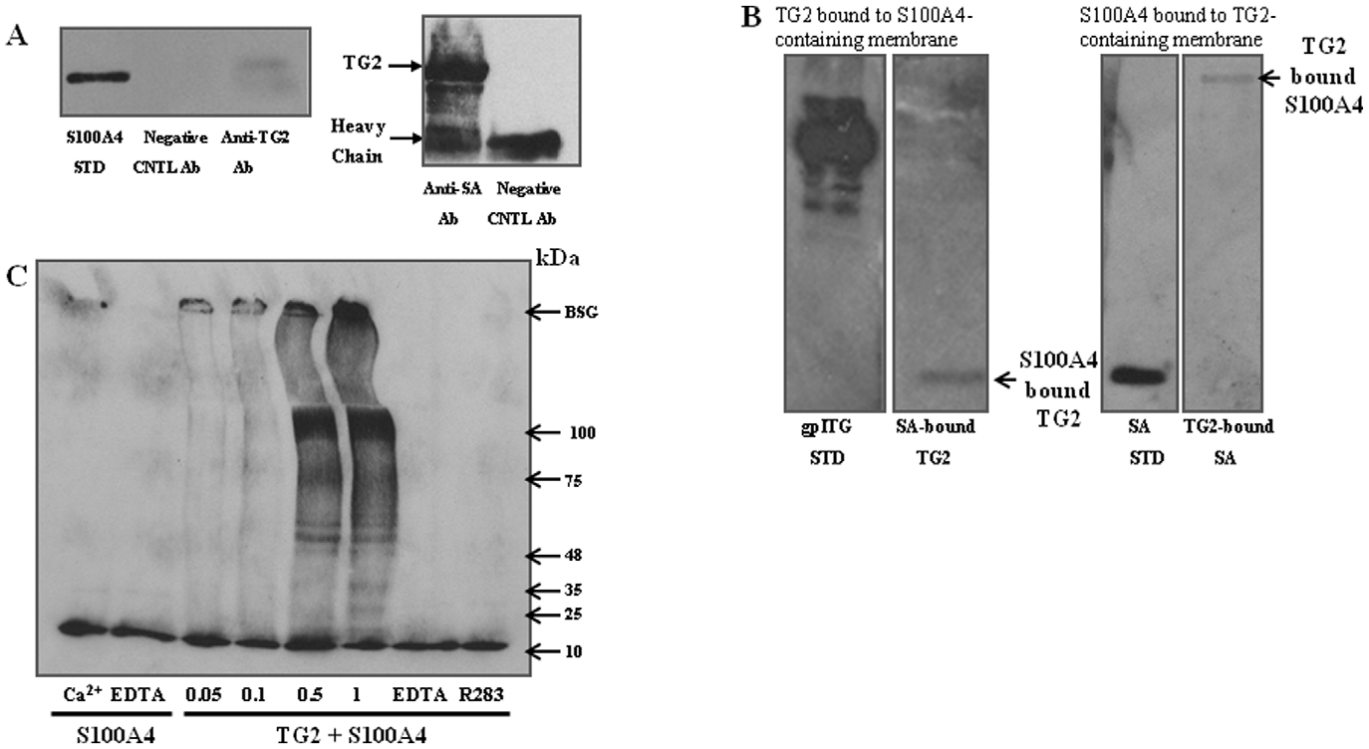
S100A4, as a substrate of TG2 also binds directly with TG2. (**A**) **Co-immunoprecipitation assay to detect the interaction between S100A4 and TG2.** After incubating recombinant S100A4 and gplTG at 37°C for 1 h, co-IP was performed. Briefly, specific antibodies, against S100A4 and TG2, respectively, were bound to the resin beads before incubation with the protein mixture to form the antibody-antigen (bait protein, S100A4 or TG2) complex. Mouse IgG and rabbit IgG were used as the negative control antibody (negative CNTL ab) for anti-TG2 antibody Cub7402 and rabbit anti-S100A4 antibody, respectively. Western blotting was carried out to detect the presence of prey proteins (TG2 and S100A4 in this case) as described in the Materials and Methods. (**B**) **The interaction between S100A4 and TG2 via Far Western blotting.** PVDF membranes bound with rhS100A4 or gplTG were prepared via Western blotting after SDS-PAGE. The membranes containing the prey protein (either S100A4 or TG2) were incubated with their relevant bait protein (gplTG or rhS100A4, respectively) as introduced in Materials and Methods and Western blotting was performed to detect the presence of the bait proteins by using their specific antibodies, i.e. Cub7402 and rabbit anti-S100A4 antibody, respectively. (**C**) ***In vitro*** **cross-linking assay to show S100A4 is a TG2 substrate.** Recombinant S100A4 (after cleavage of the his-tags) and gplTG at the concentrations in µg shown were incubated in the presence of Ca^2+^ and DTT at 37°C. The TG2 inhibitor or the Ca^2+^ chelator EDTA were used as the negative controls. The presence of S100A4 polymers was detected by using Western blotting as introduced in the Materials and Methods.

To investigate whether there is any direct interaction between TG2 and S100A4, a co-IP assay was performed by using purified TG2 and recombinant S100A4. As shown in **Fig. 6A**, by using either the specific anti-TG2 antibody Cub7402 or the anti-S100A4 antibody for the pull down and Western blotting to detect the targeted protein, the direct interaction between the two proteins can be demonstrated. Further proof of their interaction was obtained using Far Western blotting. By incubating TG2 or S100A4 (bait proteins) with the transferred membrane bound prey proteins (either S100A4 or TG2), the presence of the bait protein after binding is detected via immunoblotting (**Fig. 6B**). An *in vitro* crosslinking assay demonstrated that S100A4 is a substrate of TG2 resulting in the formation of high molecular weight polymers, that are comparable to the polymers found in the KP1 cell lysate. The formation of these crosslinked polymers could be blocked by either the addition of the Ca^2+^ chelator EDTA or the TG2 inhibitor R283 (**Fig. 6C**).

### The effect of R294 on KP1 cell morphology

**Figure 7:**
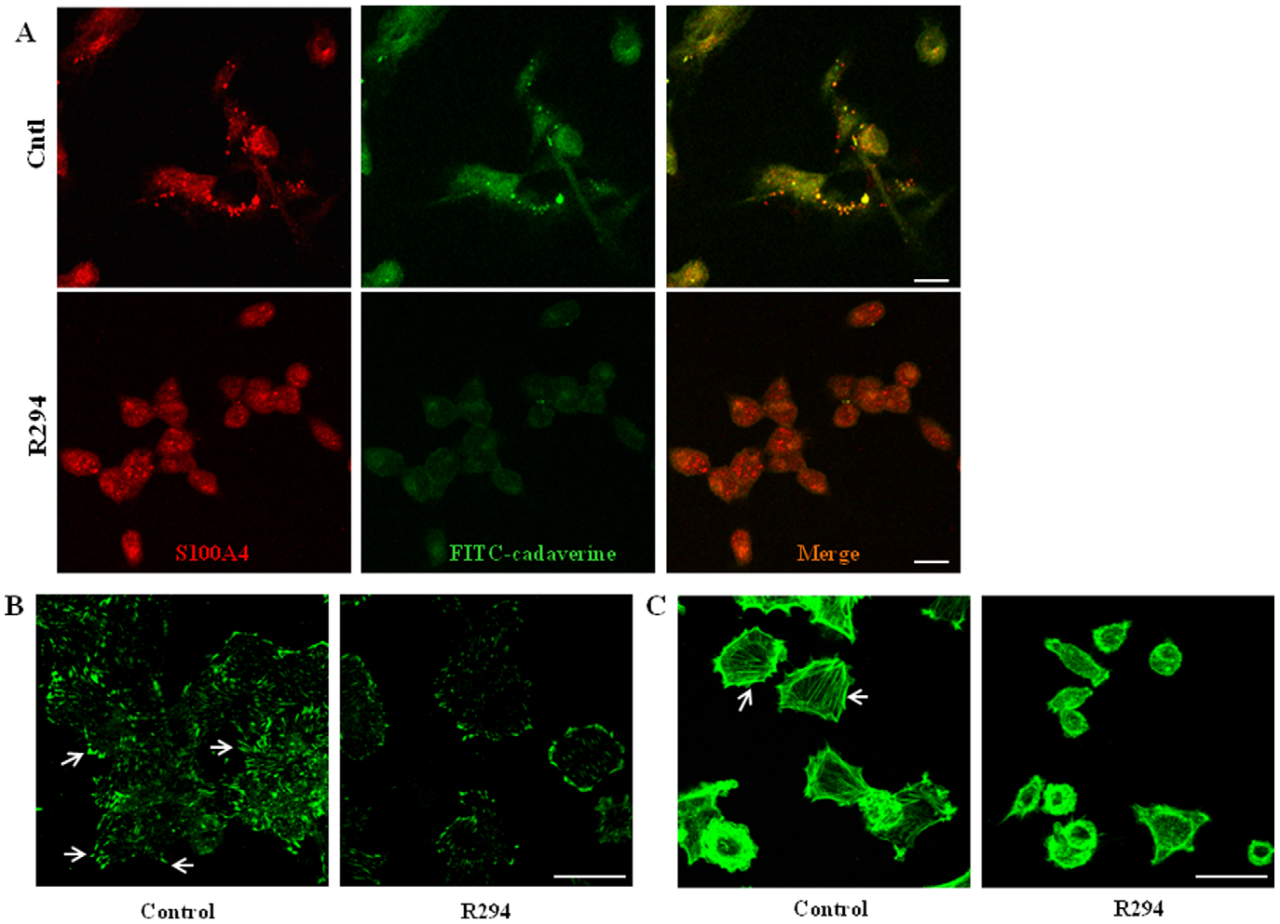
The effect of TG2 inhibitor R294 on *in situ* cell surface TG2 activity, S100A4 localisation, actin cytoskeleton and focal adhesions. (**A**) **Co-localization of** ***in situ*** **TG activity and S100A4.** *In situ* TG activity was detected via FITC-cadaverine incorporation, by incubating KP1 cells with FITC-cadaverine with or without the TG2 inhibitor R294 (0.25 mM), while extracellular S100A4 was stained by incubating the live cells with anti-S100A4 antibody as described in the Materials and Methods. Staining was visualized by confocal microscopy. Bar, 25 µm. (**B**) **The effect of TG2 inhibitor R294 on focal adhesion formation in KP1 cells.** KP1 cells were incubated with or with TG2 inhibitor R294 (0.25 mM) for 16 h and the presence of vinculin in the focal adhesions were detected via immuno-fluorescence staining by anti-vinculin antibody and a FITC-conjugated secondary antibody. Confocal microscopy was used to visualize the fluorescent signals. Bar, 35 µm. (**C**) **The effect of TG2 inhibitor R294 on KP1 actin cytoskeleton formation.** KP1 cells were incubated with or with TG2 inhibitor R294 (0.25 mM) for 16 h. Actin was stained using FITC-labelled phalloidin and the cells were visualized by confocal microscopy as described in the Materials and Methods. Bar, 35 µm.

To detect any co-localization of cell surface TG2 and S100A4 protein in the KP1 cells, fluorescence staining for *in situ* active TG2 was performed using FITC-cadaverine incorporation together with the immunofluorescent extracellular staining of the S100A4 antigen. Cells were incubated live (prior to fixing) in the presence of the antibody to S100A4 in order to immunostain the extracellular S100A4. This showed a close co-localization of the S100A4 antigen with the incorporation of FITC-cadaverine, which could be disrupted when the cell impermeable TG2 inhibitor R294 was added to the cell culture (**Fig. 7A**). Incubation of the KP1 cells with R294 also resulted in a dispersion of focal adhesion staining, when detected using vinculin (**Fig. 7B**). This dispersion of focal adhesions was accompanied by a parallel reduction in organisation of the actin cytoskeleton detected using fluorescently labelled phalloidin (**Fig. 7C**).

### Identification of the cell surface receptor(s) in regulating TG2/S100A4-mediated cell migration

**Figure 8:**
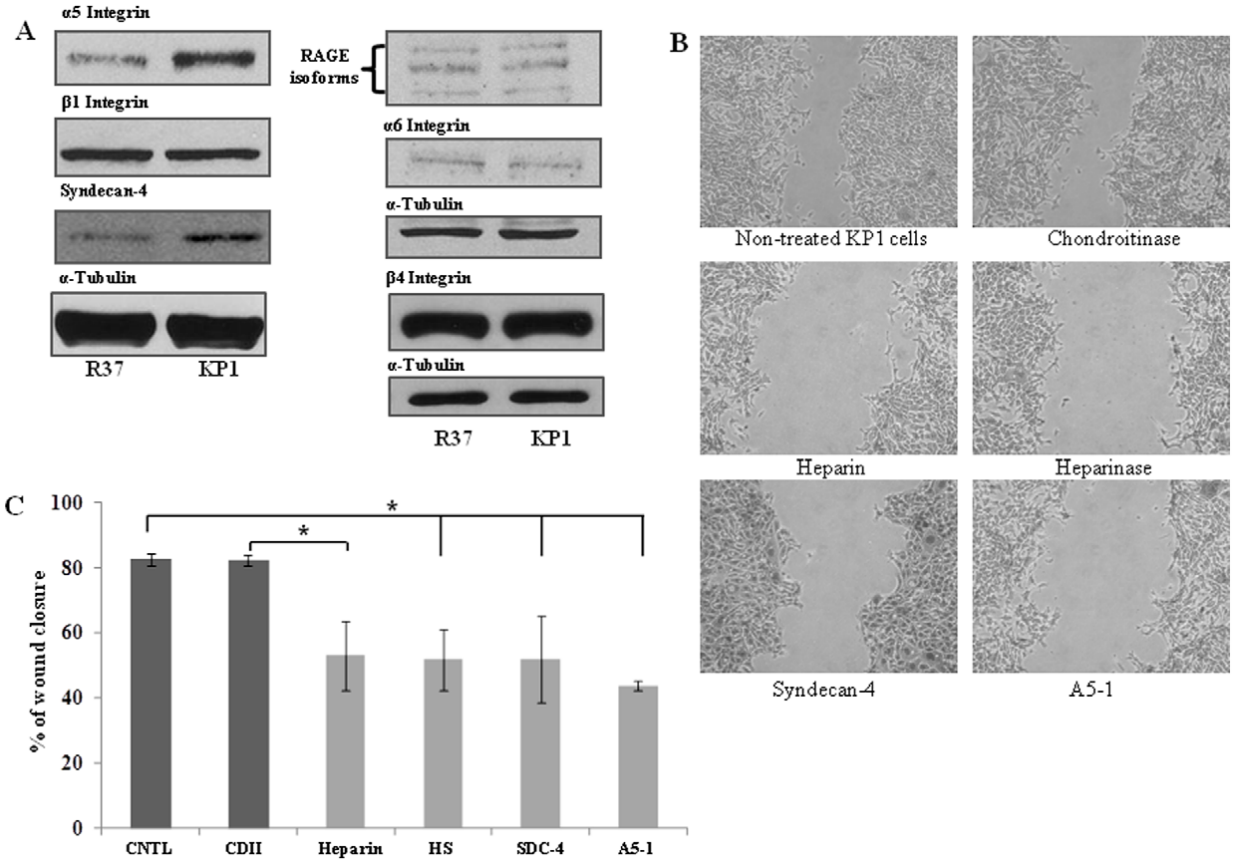
Identification of cell surface receptors in KP1 and R37 cells and their importance in KP1 cell migration. (**A**) Western blotting was performed to detect the presence of cell surface receptors in R37 and KP1 cell lysates by using their specific antibodies as introduced in Materials and Methods. α-Tubulin was used as the equal loading standard. (**B**) Wound healing assays were performed in KP1 cells treated with chondroitinase (15 mU/ml), heparinase (15 mU/ml), 0.3 mg/ml heparin, exogenous recombinant human syndecan-4 (1 μg/ml) and α5β1 integrin-targetting peptide A5-1 (5 μM) as described in the Materials and Methods. At least 3 separate experiments were performed per treatment. And the mean percentage wound closure ± S.D. shown in (**C**). *, p<0.05 between the groups as indicated in the figure.

Cell surface receptor(s) that extracellular S100A4 and TG2 are reputed to be associated with and in turn can regulate cell migration were detected via Western blotting. Increased expression of both syndecan-4 and α5 integrin was detected in KP1 cells when compared to R37 cells although present in both cells, while no difference in expression of other receptors, including RAGE, β1, α6 and β4 integrins, was found between R37 and KP1 cells (**Fig. 8A**). **Fig. 8B** shows the different approaches used to functionally block these cell surface receptors and the observed effect on the migratory ability of KP1 cells. Addition of heparin (which can compete with cell surface heparan sulphates for the binding of TG2 or S100A4 protein) or heparinase (which digests the cell surface heparan sulphate chains) to KP1 cells was found to significantly block cell migration, suggesting the involvement of cell surface heparan sulphates in this process. Importantly, by adding recombinant syndecan-4 to KP1 cells to compete out the effect of cell surface syndecan-4, it was shown that addition of this exogenous protein also significantly inhibited the migration of KP1 cells (**Fig. 8B** and **C**). This strongly suggests that the candidate cell surface heparan sulphate is syndecan-4, which is also the major syndecan found in focal adhesions In addition the α5β1 integrin targeting A5-1 peptide which is important in TG2/syndecan-4 mediated cell adhesion [16] also significantly blocked the S100A4-enhanced cell migration in KP1 cells (**Fig. 8B** and **C**).

### S100A4 can function extracellularly and cell surface S100A4 is crosslinked by TG2, both of which can be blocked with TG2 inhibitors

**Figure 9:**
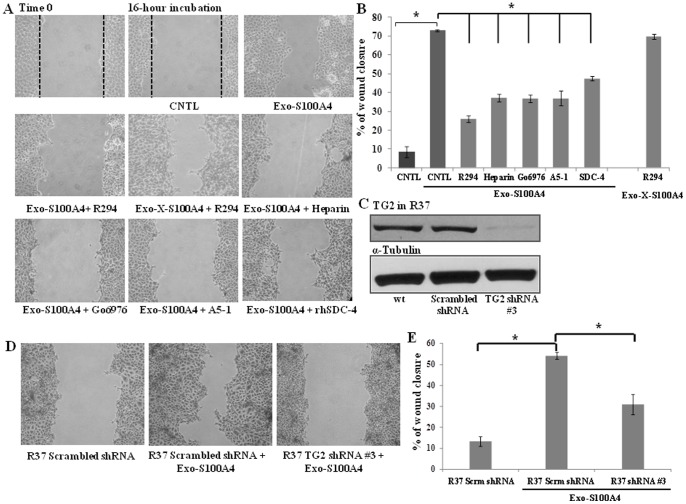
Exogenous S100A4 enhances cell migration in R37 cells, which can be blocked by TG inhibitors and agents that interfere with syndecan-4 signalling. (**A**) The wound healing assay in R37 cells was carried out in the presence of extracellular recombinant S100A4 (exo-S100A4,1 μg/ml) with or without TG2 inhibitor R294 (0.25 mM) or with TG2 crosslinked-S100A4 in (exo-X-S100A4) in the presence of R294. The wound assay was also carried out in the presence of heparinase (15 mU/ml), exogenous recombinant human syndecan-4 (1 μg/ml), α5β1 integrin-targetting peptide A5-1 (5 μM) and PKCα inhibitor Go6976 (5 μM) added in the presence of exogenous S100A4 as described in the Materials and Methods. At least 3 images were taken from each wound to verify the closure rate of the wound and three separate experiments were performed. (**B**) Shows the mean percentage wound closure mean ± S.D. from 3 separate experiments. *, p<0.05 between the groups as indicated in the figure. (**C**) **Knocking down of TG2 in R37 cells.** Western blotting was performed to detect TG2 in R37 stably transfected with TG2 shRNA #3 or the scrambled shRNA control. (**D**) Wound healing assay was carried out on R37 cells transfected with scrambled or TG2 shRNA #3 in the presence of exogenous S100A4 treatment and analysed (**E**) As described in the Materials and Methods. *, p<0.05 between the groups as indicated in the figure.

Given the cell surface co-localization of TG2 activity and S100A4, the ability of the non-cell permeable TG2 inhibitors R281 and R294 to reduce this and in parallel inhibit KP1 cell migration, our results suggest that the major action of TG2 on S100A4 is likely to be through an extracellular mediated signalling pathway involving the cell surface receptor syndecan-4. To test this hypothesis, recombinant S100A4 protein was first used to exogenously treat the control R37 cells. As shown in **Fig. 9A** and **B** the addition of exogenous S100A4 promoted the migration of R37 cells. To confirm that TG2 was also involved in this exogenous S100A4 stimulated migration cells were incubated with the TG2 inhibitor R294, which resulted in significant inhibition of the S100A4 stimulated migration (**Fig. 9A** and **B**). Importantly S100A4 crosslinked *in vitro* and then added back to the inhibited R37 cells could partially compensate the effect of TG2 inhibitor R294 (**Fig. 9A** and **B**). To further confirm that the effect of the exogenously added S100A4 shares a similar signalling pathway to that of the KP1 cells which over-express endogenous S100A4, wound healing assays were performed. Following cell wounding, heparinase, recombinant syndecan-4, A5-1 inhibiting peptide against α5β1 integrins and PKCα inhibitor Go6976 were added to the scratched cells resulting in significant inhibition of the exogenously stimulated S100A4 migration (**Fig. 9A** and **B**). The knock down of TG2 expression by shRNA (#3) in R37 cells (**Fig. 9C**), which had no effect on the key receptors β1 and α5 integrin or Syndecan-4 when compared to their scrambled shRNA controls (**Fig. S1**), also led to the failure of the cells to respond to the exogenous S100A4 treatment in the wound healing assay (**Fig. 9D** and **E**), confirming the involvement of TG2 in extracellular S100A4-induced cell migration.

### The involvement of PKCα and syndecan-4 in TG2 and S100A4-related signalling

**Figure 10:**
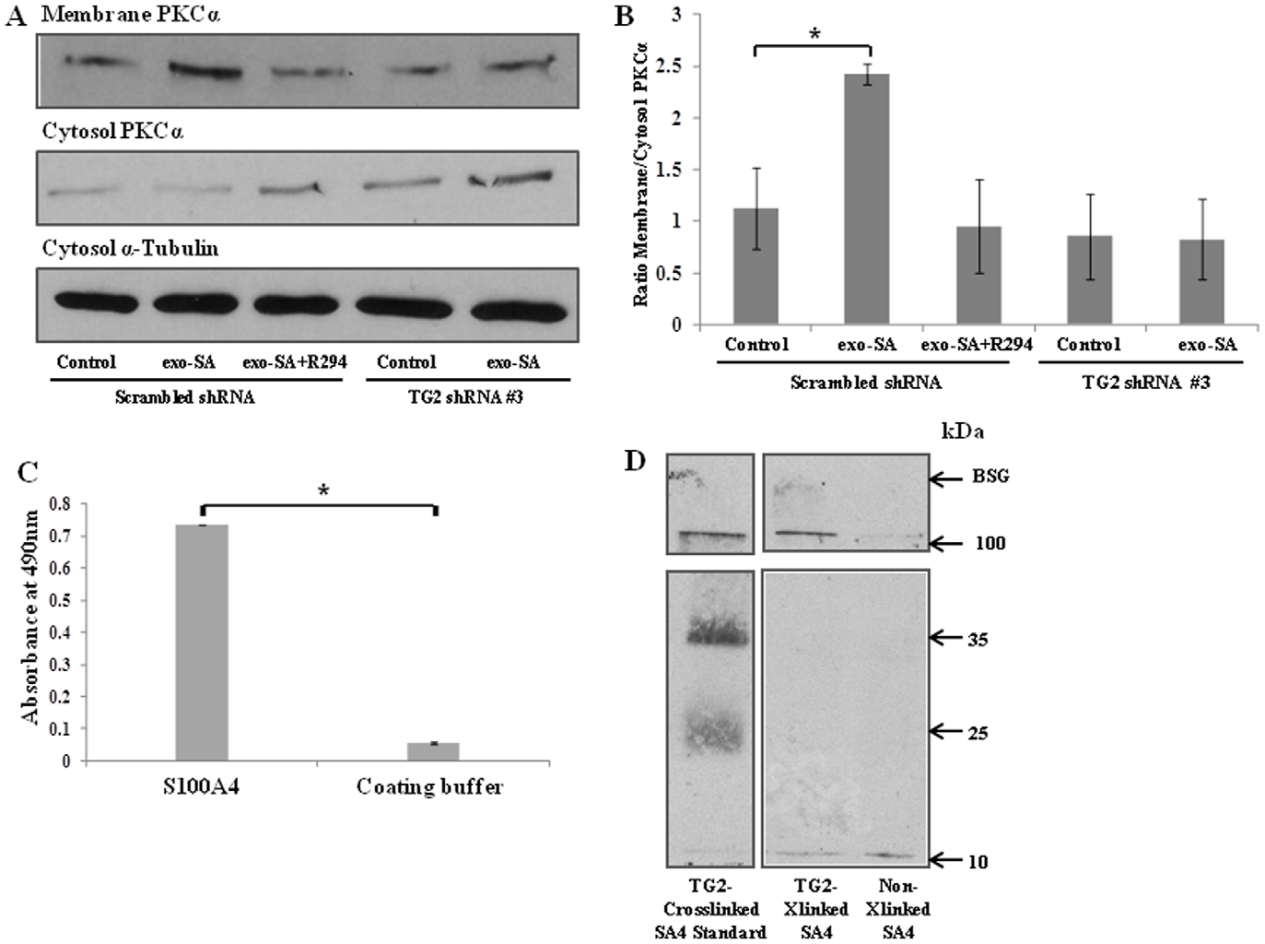
The involvement of syndecan-4/PKCα. (**A**) The activation of PKCα. The presence of PKCα in the cytosol and membrane fractions, after exogenous S100A4 treatment of R37 cells transfected with scrambled or TG2 #3 shRNA, or R37 cells treated with R294 was detected using Western blotting as introduced in the Materials and Methods. (**B**) Densitometry was performed to measure the Western blots from 3 separate experiments and the ratio of membrane bound to cytosol PKCα plotted as a ratio after normalisation of the cytosol PKCα for protein loading *, p<0.05 between exogenous S100A4 treated and the non-treated R37 cells transfected with the scrambled shRNA. (**C**) **The direct interaction between syndecan-4 and S100A4 via co-IP.** Recombinant human S100A4 (after cleavage of the his-tags) was crosslinked by TG2 as introduced in Materials and Methods. The crosslinked S100A4 was incubated with recombinant syndecan-4 at 37°C for 1 h and co-IP was performed using anti-syndecan-4 antibody and Western blotting was performed to detect the presence of S100A4 as described in the Materials and Methods. The non-crosslinked S100A4, namely the S100A4 protein incubated with inactive TG2 and syndecan-4 was used as the control group. (**D**) **Solid binding assay showing interaction between S100A4 and syndecan-4.** The binding assay was performed to detect the interaction between S100A4 (pre-coated onto 96 well plates after cleavage of the his-tags) and recombinant human syndecan-4 as detailed in the Materials and Methods. The coating buffer was used as the negative control coating. *, p<0.05 between the groups.

The functional link between the activation of PKCα and TG2/S100A4 stimulated migration was studied by detecting the translocation of activated PKCα from the cytosol fraction to the membrane. As shown in **Fig. 10A** and **B** exogenous S100A4 treatment of R37 cells increased the presence of the PKCα in the membrane fraction, which was inhibited by the cell impermeable TG2 inhibitor R294. Importantly, reduced membrane PKCα was also found in the TG2 shRNA-transfected R37 cells, which failed to respond to the exogenous S100A4 treatment. To further investigate the direct association between syndecan-4 and S100A4, a solid phase binding assay was first performed, which showed the binding of syndecan-4 to the immobilized S100A4 (**Fig. 10C**). Another approach of *in vitro* co-IP was applied to confirm the direct interaction between syndecan-4 and TG2 crosslinked S100A4 by using *in vitro* crosslinked or non-crosslinked S100A4 incubated with syndecan-4. Western blotting was then used to detect the presence of S100A4 in the syndecan-4-related immuno-complex (**Fig. 10D**). A strong signal for S100A4 polymers was found in the syndecan-4 immunocomplex pulled down by anti-syndecan-4 antibody, while a lesser amount of S100A4 monomer was found bound to the syndecan-4 protein.

### The role of S100A4 in TG2-regulating breast cancer migration

**Figure 11:**
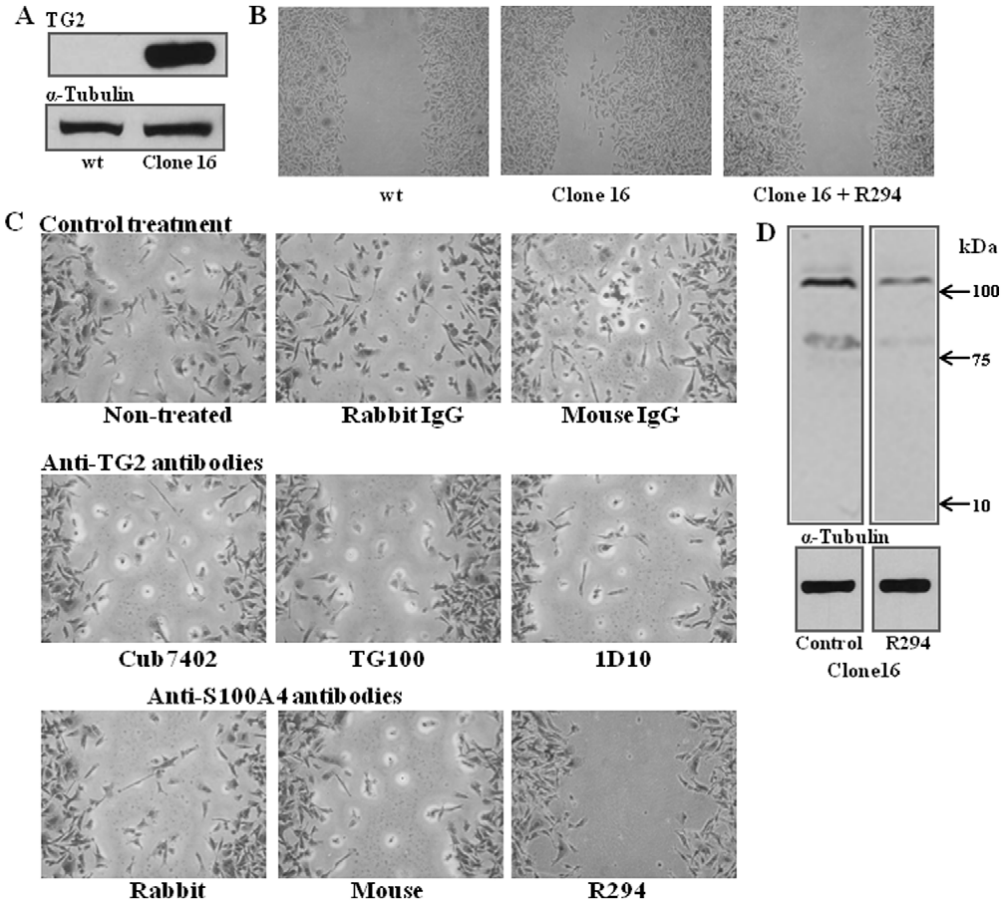
The role S100A4 in TG2-related breast cancer cell migration. (**A**) **The presence of TG2 in MDA-MB-231 cell lines**. Western blotting was performed to detect TG2 in MDA-MB-231 wild type and clone 16 cells. (**B**) **Wound healing assays for clone 16 and wild type MDA-MB-231 cells with or without treatment with TG2 inhibitor R294.** (**C**) **The importance of TG2 and S100A4 in MDA-MB-231 breast cancer cell migration.** MDA-MB-231 wild type and clone 16 cells were used in the wound healing assay in the presence of TG2 antibodies (Cub7402, TG100 and 1D10) and anti-S100A4 antibodies (rabbit polyclonal and mouse monoclonal) at the concentrations of 20 μg/ml, while TG2 inhibitor R294 was used at the concentration of 0.25 mM. Mouse control IgG and rabbit control IgG were used as the control treatments. At least 3 images were taken from each wound to analyse the closure rate of the wound and three separate experiments were performed. (**D**) **S100A4 in MDA-MB-231 clone 16 cells.** Western blotting was performed to detect the presence of S100A4 antigen in clone 16 cells in the presence or absence of TG2 inhibitor R294 by using specific anti-S100A4 antibody.

The human breast cancer cell line MDA-MB-231 was used to extend our observation for the importance of both TG2 and S100A4 proteins in R37 and KP1 cells to human cancer cells. Higher TG2 expression was detected in the clone 16 cells, while negligible TG2 was found in the wild type MDA-MB-231 cells (**Fig. 11A** **)**. In the wound healing assay, increased migration was observed in the high TG2 expressing clone16 cells when compared to the wild type MDA-MB-231 cells which contain very low levels of TG2 (**Fig. 11B**). Importantly in addition to R294 the TG2 monoclonal antibodies Cub7402, TG100 and 1D10 and the polyclonal antibody for S100A4 could block the migration of these cells. As found with KP1 cells, this suggests the involvement of extracellular TG2 and S100A4 in regulating MDA-MB-231 clone 16 cell migration (**Fig. 11C**). Western blotting for S100A4 antigen in clone16 cells showed the presence of high-molecular weight S100A4 polymers, comparable to those found in the *in vitro* crosslinking assay and in the KP1 cells, which could be reduced by treatment of cells with the non-cell permeable TG2 inhibitor R294 (**Fig. 11D**).

## Discussion

S100A4, acting as either an intracellular or extracellular protein, can promote cell migration using a number of different pathways, such as the activation of MMPs [30] through integrin receptors [6] or through the RAGE signalling pathway [5], but the exact mechanism is still not fully understood. Another protein shown to promote cell migration is TG2, which can function extracellularly via its binding to FN and heparan sulphate proteoglycans. Over-expression of both proteins in cancer cells has been linked to tumour progression [2]. In this paper we used the well-characterised R37 cells [20] transfected with S100A4 to assess whether the functions of TG2 and S100A4 are interrelated. Investigation of TG2 activity in R37 control cells (transfected with empty vector) and highly-metastatic KP1 cells (Rama cells transfected with S100A4) indicated the presence of enzyme activity in the cell lysates and on the cell surface. Interestingly, the amount of TG2 found at the cell surface was greater in the KP1 cells, even though the amount of TG2 protein found in the cell lysates was slightly greater in the R37 cells. However, such a redistribution of enzyme is not unusual under pathological situations [31]. The first indication of a link between S100A4 and TG2 activity was obtained by treatment of the metastatic KP1 cells with specific site-directed irreversible inhibitors of TG2, R281, R283 and R294, and the competitive primary amine substrate MDC, which were each able to block cell migration of the S100A4 transfected KP1 cells. The finding that the cell impermeable inhibitors, R281 and R294, inhibited cell migration suggested that the involvement of TG2 in the migratory process is likely to be at the cell surface in the KP1 cells. Moreover, the inhibitory effect of MDC suggested that it is the transamidation activity of TG2 that functions in S100A4-mediated cell migration, instead of a conformation change, which can be induced by the binding of site-directed irreversible TG2 inhibitors [32]. Importantly knockdown of the expression of TG2 in KP1 cells abolished its migratory capability, further confirming the involvement of TG2 in S100A4-related migration.

As a consequence of the finding that the extracellular S100A4 can enhance cell migration [33], our next step was to add S100A4 to the control R37 cells, which led to stimulation of migration and which could be blocked by the cell impermeable TG2 inhibitor R294. Migration could also be blocked by down-regulation of TG2 expression by shRNA. Hence our data suggest that in these cells both TG2 and S100A4 are acting extracellularly to stimulate cell migration. In order to further study the relationship between TG2 and S100A4 in regulating cell migration, a series of *in vitro* studies were initially performed using pure proteins. Co-IP assays revealed the direct interaction between these two proteins, while *in vitro* cross-linking assays indicated that S100A4 could act as a TG2 substrate, as reported for other members of the S100A family [17]. TG2 (using FITC-cadaverine incorporation) and immunostaining for the extracellular S100A4 antigen in KP1 cells indicated the extracellular co-localization of the enzyme with the protein, which could be inhibited by incubation of cells with the cell impermeable TG2 inhibitor R294. Incubation of KP1 cells with R294 also resulted in the disassembly of the punctuate cell surface staining of S100A4 in these cells. Given the possibility that S100A4 is associated with focal adhesions, it seems feasible that the parallel disassembly of the actin cytoskeleton organization and focal adhesions in the presence of the extracellular acting TG2 inhibitor are interrelated. Importantly the assembly of focal adhesions and the actin cytoskeleton is essential for cell migration. This data also supports the role of S100A4 in regulating actin cytoskeleton re-organization [1], however in this case the action on the actin cytoskeleton seems to be mediated by an extracellular event which involves TG2. The importance of S100A4 polymers in enhancing cell migration has been well reported. It was therefore important to show that add back of *in vitro* crosslinked S100A4 to TG2 inhibited R37 cells (i.e. the cells treated with TG2 inhibitors) could restore cell migration. The demonstration of this rescuing effect of TG2 crosslinked S100A4 further confirmed that cell surface TG2 activity regulates extracellular related S100A4-mediated cell migration via its crosslinking.

It has been shown that cell surface receptors, such as RAGE or α6β4 integrin, are required by S100A4 to regulate cell migration [1]. TG2, when bound to FN, can mediate cell adhesion either via acting as a co-receptor for β1 and β3 integrins [10] or via mechanism involving the direct binding to syndecan-4 and activation of α5β1 via PKCα [23], [34]. Interestingly recent reports also indicated the importance of heparan sulphates in S100A4-relevent cell migration [7]. To study the signalling pathway in TG2-regulated cell migration by S100A4, Western blotting was used to reveal any changes in cell surface receptors in the S100A4- transfected cells, which revealed comparable receptor profiles although slightly increased expression of syndecan-4, α5 integrins in KP1 cells when compared to R37 control cells. No changes were found in the expression of RAGE and α6β4 integrin. The involvement of α5β1 integrin in the migratory mechanism of KP1 or S100A4-treated R37 cell was confirmed by the ability of the α5β1 integrin-targeting peptide A5-1 [16] to inhibit cell migration in the wound healing assay. The importance of cell surface heparan sulphates in facilitating the S100A4-regulated cell migration was demonstrated by the exogenous addition of heparin, heparinase or recombinant syndecan-4 protein to cells, which significantly slowed down cell migration. This strongly suggests that syndecan-4 is the major heparan sulphate proteoglycans involved in S100A4 related migration. Moreover, disruption of both focal adhesion assembly by the TG2 inhibitor R294 and the parallel disruption of the punctate staining pattern of S100A4 at the cell surface suggest that S100A4 is found associated with syndecan-4 present in focal adhesions, thus explaining our observations and link between the re-organization of focal adhesions and TG2 inhibition.

Since syndecan-4 and α5β1 integrin mediate cell migration via the activation of PKCα [23], [34], it was not surprising to find that both the PKCα inhibitor Go6976 and the GK21 peptide (which is reported to block the direct interaction between intracellular domain of β1 integrin and PKCα [23], [35]) blocked the migration of KP1 cells and S100A4-stimulated R37 cells. This suggests that PKCα could be the important intracellular signalling link in TG2 and S100A4-mediated cell migration, since it has been shown that PKCα is the signalling link between α5β1 integrin and syndecan-4 co-signalling [16], [23]. By studying the translocation of PKCα from cytosol to membrane, an indication of its activation, we showed enhanced translocation of PKCα in the exogenous S100A4-treated R37 cells, which was inhibited by TG2 inhibitor R294. Importantly, R37 cells transfected with TG2 shRNA showed lower levels of active PKCα and failed to respond to S100A4 treatment. This data provides the direct link between TG2 and S100A4-related cell migration, which is through a syndecan-4/α5β1 integrin co-signalling pathway linked by PKCα. A series of further *in vitro* studies confirmed the direct interaction between sydecan-4 and S100A4 as shown by using a solid binding assay and co-IP. It is shown that there is a stronger binding between crosslinked S100A4 polymers and syndecan-4, compared to the non-crosslinked S100A4 monomer, further supporting our finding that transamidation of S100A4 is a prerequisite to its biological action, which is also confirmed by the ability of crosslinked S100A4 to partially compensate the effect of TG2 inhibitor in R37 cell migration. Since S100A4 and TG2 are also thought to play an important role in the progression of a number of human cancers including breast cancer [2], [36], it was important to test our theory in a breast cancer cell system [37]. Hence for this study we used a well-characterised breast cancer cell line MDA-MB-231 clone 16, which has been shown to have very high TG2 expression, is more migratory and more resistant to doxorubicin, compared to its wild type cell line [15]. These cells contain high molecular weight immuno-reactive polymers of S100A4, which can be partially decreased by treatment with the TG2 inhibitor R294. Interestingly, the reduction of S100A4 polymer formation by R294 did not restore the presence of the S100A4 monomer. The S100A4 monomer was also not detectable in the non-treated clone 16 cells. A possible explanation for this is that the high TG2 levels in non- treated clone 16 cells maintains the S100A4 protein in its crosslinked form, thus preventing it from degradation and prolonging its effect on regulating the metastastic phenotype. In such a situation, the inhibition of polymer formation by R294 would also lead to the rapid degradation of any resulting non-crosslinked S100A4 monomer, thus accounting for its absence when polymer formation is partially inhibited. This protective effect of protein crosslinking by transglutaminases is well recognised and widely reported [38], [39]. Importantly this reduction in S100A4 polymer content is paralleled by an inhibition of cell migration in these cells when exposed to the TG2 inhibitor R294 and to antibodies directed against either TG2 or S100A4. Hence the involvement of TG2 with S100A4 in mediating the increased migration found in a rat mammary cancer cell line may also be extended to human breast cancer cells.

**Figure 12:**
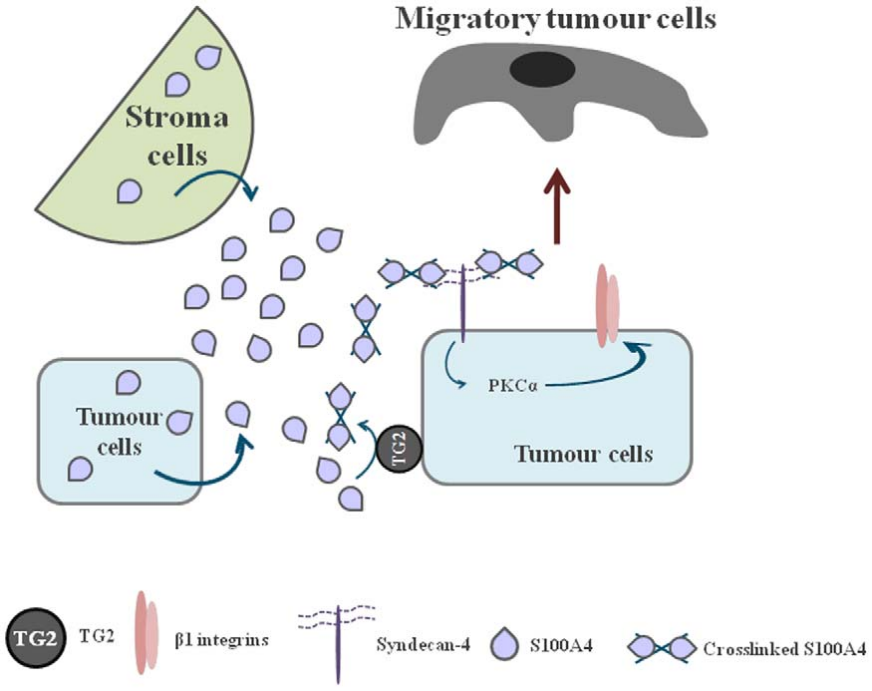
Diagram showing the potential role of TG2 in S100A4-regulated cell migration. The secretion of S100A4 from both tumour stromal and tumour cells themselves can be cross-linked by cell surface/matrix TG2. Once crosslinked by TG2, S100A4 shows a high binding affinity for heparan sulphate chains of syndecan-4 on the tumour cell surface and activates the syndecan-4 and inside out α5β1 integrin co-signalling pathways via the intracellular signalling molecule PKCα, which accelerates tumour cell migration and metastasis.

In conclusion, for the first time, our work demonstrates the crucial link between two well-known important bio-markers for highly metastastic cancers, namely TG2 and S100A4. We have now shown that S100A4 is a substrate of TG2. That crosslinking of S100A4 by TG2 can be inhibited by the TG2 specific inhibitor R294, leading to the abolishment of S100A4-enhanced cell migration. Importantly we demonstrate the mechanism for this S100A4/TG2-related cell migration involving the activation of the syndecan-4 and α5β1 integrin co-signalling pathway linked by PKCα (**Fig. 12**). Our data reveal a novel potential strategy in cancer therapy, which is to antagonize the link between TG2 and S100A4 by inhibition of the crosslinking activity of TG2. By discovering the mechanism in this signalling transduction pathway, we propose that a combined treatment of TG2 inhibition with the inhibition of key signalling targets e.g. PKCα could be more promising therapeutic approach for the treatment of S100A4 and TG2 containing tumours.

## Supporting Information

Figure S1 The presence of cell surface receptors in the shRNA transfected R37 and KP1 cells. Western blotting was performed to detect the presence of α5, β1 integrins and syndecan-4 in the TG2 shRNA #3 transfected R37 cells (A) or KP1 cells (B), while the scrambled shRNA transfected cells were used as the control.
(PDF)
