# Supplementary material for: The Role of TG2 in Regulating S100A4-Mediated Mammary Tumour Cell Migration
Source: PLoS One. 2013 Mar 1;8(3):e57017. doi: 10.1371/journal.pone.0057017 (PMC3585722; doi:10.1371/journal.pone.0057017)
Supplement: Figure S1 — The presence of cell surface receptors in the shRNA transfected R37 and KP1 cells. Western blotting was performed to detect the presence of α5, β1 integrins and syndecan-4 in the TG2 shRNA #3 transfected R37 cells (A) or KP1 cells (B), while the scrambled shRNA transfected cells were used as the control. (PDF) [file pone.0057017.s001.pdf]

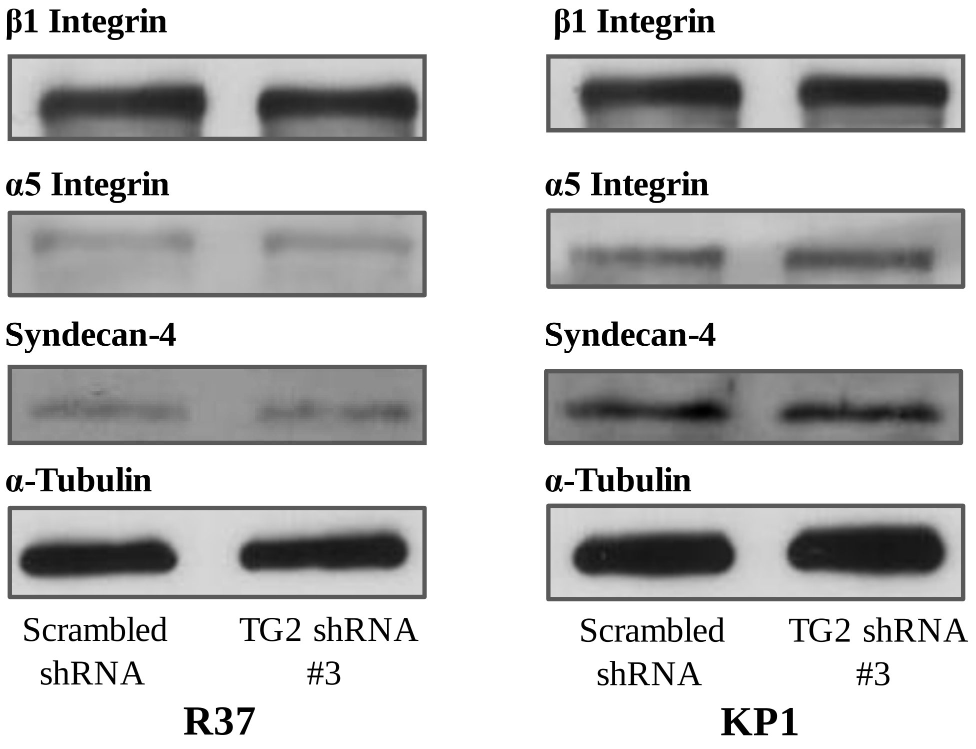

Figure S1

Fig. S1. The presence of cell surface receptors in the shRNA transfected R37 and KP1 cells. Western blotting was performed to detect the presence of the cell surface receptors in R37 and KP1 cells stably transfected with TG2 shRNA, while the scrambled shRNA transfected cells were used as the control as described in Materials and Methods.
